# Randomized controlled expressive writing pilot in individuals with Parkinson’s disease and their caregivers

**DOI:** 10.1186/s40359-015-0101-4

**Published:** 2015-11-30

**Authors:** Therese Verkerke Cash, Sarah K. Lageman

**Affiliations:** Parkinson’s and Movement Disorders Center, Virginia Commonwealth University, P.O. Box 980539, Richmond, VA 23298-0539 USA; Department of Psychology, Virginia Commonwealth University, Richmond, USA; Department of Neurology, Virginia Commonwealth University, Richmond, USA

**Keywords:** Expressive writing, Salivary cortisol, Parkinson’s disease, Quality of life, Caregiver

## Abstract

**Background:**

Individuals with Parkinson’s disease (PD) and their caregivers are at risk for emotional distress and hypercortisolism. Expressive writing is an effective complementary intervention to ameliorate the psychological and physiological effects of chronic illness. This pilot study aimed to evaluate feasibility and preliminary effectiveness of an expressive writing intervention for individuals with PD and their caregivers.

**Methods:**

Individuals with PD (*N* = 27) and their caregivers (*N* = 14) were randomly assigned to expressive (*N =* 15 patients, eight caregivers) or neutral (*N =* 12 patients, six caregivers) writing conditions. Cortisol awakening response (CAR), non-motor functioning, quality of life, and performance on tests of cognitive functioning were assessed at baseline, immediate post, 4-month, and 10-month post intervention.

**Results:**

Attrition was a challenge as eight patients (29.62 %) and four caregivers (28.57 %) chose to discontinue before beginning the intervention or were lost to follow up prior to completing the intervention or the first follow up visit. Significant reduction in anxiety, marginally significant improvement in depression and caregiver burden, and significant improvements in performance on tests of learning and memory were observed, but these changes did not differ by writing condition. CAR significantly differed over time between patients and caregivers and writing conditions.

**Conclusions:**

Some evidence for the feasibility and effectiveness of writing to alleviate hypercortisolism was demonstrated in a small sample of PD patients; however, relatively high attrition rates and the lack of difference between expressive and neutral writing conditions on emotional and neurocognitive outcomes suggests expressive writing procedure modifications may be needed to obtain optimal results for this population.

**Trial registration:**

ClinicalTrials.gov, NCT02217735, Study Start Date: August 30, 2011.

**Electronic supplementary material:**

The online version of this article (doi:10.1186/s40359-015-0101-4) contains supplementary material, which is available to authorized users.

## Background

Expressive writing is a brief psychosocial intervention with known emotional and physical health benefits [16]. The classic expressive writing paradigm [37] involves 3, 20 min writing sessions within a 2 week period, during which participants are encouraged to openly express their thoughts and feelings about a stressful or traumatic real life event. Research supports the use of expressive writing as an adjunctive therapy in chronically ill populations [2]. Specifically, expressive writing has led to both mental and physical health gains in patients diagnosed with fibromyalgia [5], asthma and rheumatoid arthritis [47], and cancer [54]. Additionally, a form of expressive writing has been successfully employed to reduce depressed mood in chronic pain patients [19], to decrease cortisol reactivity and enhance post-traumatic growth in PTSD patients [48], and to moderate autonomic response in individuals with elevated blood pressure [35].

Individuals with Parkinson’s disease (PD) and their caregivers are at risk for increased emotional distress [1], cognitive declines [51], and elevated cortisol levels [10, 12, 22]. While research on psychosocial treatment options in PD are limited, a recent randomized clinical trial compared cognitive-behavioral therapy (CBT) to usual care in a sample of individuals with PD and found improvements in symptoms of depression, anxiety, quality of life (QOL), coping, and PD symptom ratings for the CBT group only, and these benefits were sustained at a 14-week follow up assessment [13]. Caregivers of individuals with PD also showed improvement on measures of caregiver strain and subjective burden after receiving 12–14 sessions of CBT compared to those in a no-treatment control group [44].

While CBT seems to be effective, many individuals may be unwilling or unable to partake in weeks of therapy to reduce physical and emotional stress. Therefore, it may be fruitful to consider briefer, cost effective alternatives. Expressive writing is one such alternative therapy with strong empirical support in other related groups that may enhance physical and emotional functioning in patients and caregivers coping with PD. It has also been found to augment working memory through reductions in avoidant and intrusive thoughts [28]. Expressive writing has been theorized to operate according to some of the same principles as CBT (i.e., exposure, cognitive restructuring) [45], and it has been demonstrated to consistently result in small to moderate effect sizes [16, 46].

The use of expressive writing in PD may be particularly well-suited to this population due to findings that both PD and caregiving are associated with abnormalities in stress hormone levels. One study [22] found evidence of sustained hypercortisolism in PD patients compared to normal controls. More recent studies have linked high cortisol levels to behavioral problems in PD [12] and elevated cortisol levels upon awakening in individuals caring for dementia patients [10]. Negative consequences of hypercortisolism include detriments to multiple organ systems and declines in cognition and memory systems [22].

Therefore, efforts to reduce cortisol levels through psychosocial interventions are likely to have important implications for overall physical and emotional health in individuals with PD and their caregivers. For example, one recent study showed reductions in cortisol concentration in a sample of PD patients following both an active therapeutic massage intervention and a control resting to music condition [50]. The cortisol awakening response (CAR), captured by assessing cortisol concentrations at intervals after waking, is a metric of HPA axis activation that is associated with psychosocial functioning [8] and has been shown to be a valuable measure for assessing hyper- and hypocortisolemic traits in patients with depression and chronic fatigue [24].

Expressive writing is a novel psychosocial intervention that may effectively target non-motor symptoms and emotional and physical stress and enhance QOL for patients and caregivers coping with PD. Based on the existing literature, we anticipate that an expressive writing intervention will lead to improvements in self-reported emotional functioning and cognitive performance, and reductions in objective stress hormone levels among individuals with PD and their caregivers and that these benefits will be sustained at 4-month and 10-month follow up evaluations. Primary outcomes of this study include self-reported anxiety, depression, apathy, non-motor symptoms (PD patients only), health-related QOL (PD patients only), overall QOL, caregiver burden (caregivers only), performance on cognitive tests assessing processing speed, auditory attention, learning, memory, mental flexibility, and working memory, and CAR.

## Methods

### Participants

In this pilot study, individuals with PD (*N* = 27) and their caregivers (*N* = 14) were recruited and randomly assigned to expressive (*N =* 15 patients, eight caregivers) or neutral (*N =* 12 patients, 6 caregivers) writing conditions. Participants were patients or family members of patients recruited from a movement disorders specialty clinic in the southeastern United States during routine clinical exams or through the clinic’s email listserv. Inclusion criteria for patients included a neurologist-confirmed diagnosis of PD and adult age (18 or older). Adult age caregivers were only able to participate if the individual with PD that they care for participated in the study. Patients were excluded if their clinical records showed evidence of a severe, untreated mental illness and/or a dementia diagnosis. Study activities were completed from August 2011 until June 2013. Recruitment for the study was ended in order to conclude data collection within an approximately 2 year timeframe. Initially, 66 individuals with PD were identified as meeting inclusion criteria for this study, and all were contacted by phone or in person to discuss participation. Of those initially identified as eligible, 39 opted not to participate, citing travel distance to the clinic, other time commitments, or a lack of interest as their reasons for declining. (Please see Additional file 1: Figure S1 in supplemental materials for CONSORT flow diagram of study recruitment and retention.) No significant differences on demographic characteristics were noted between those who elected to participate and those who declined. Mean age of the entire sample was 67.15 (*SD* = 7.63). The sample was equally divided among males and females (50 % female). The average level of education was 16 years. All participants identified as White and not of Hispanic origin. No significant differences on these sample characteristics were found across the two writing conditions or between patients and caregivers. This study was completed with adequate understanding and written consent of the participants and with the ethical approval of the Virginia Commonwealth University Institutional Review Board. Participants were given the option to participate with or without providing salivary cortisol samples.

### Design

This study employed a 2 Writing Condition (expressive writing, neutral writing) x 2 Participant Status (Patients, Caregivers) x 4 Time (Baseline, Immediate Post, 4 Month, 10 Month) mixed design. Participants were randomly assigned to an experimental (expressive writing) or a control (neutral writing) condition (see instructions below). Patients and caregivers who participated in the study together were assigned to the same writing condition but completed the writing exercises independently. The decision to assign patients and caregivers to the same writing condition was based on two assumptions. First, we predicted that patients and caregivers participating together would be likely to discuss the intervention and that assigning them to different conditions might dilute the effects of the writing condition to which they were assigned. Second, we assumed that effects of the intervention on one participant in the dyad might affect the other such that assigning participants within a dyad to different writing conditions could have added additional error variability for which we were unable to control in a small pilot study. Randomization order was generated using an online randomization generator (http://www.randomizer.org/), and participants were assigned to condition in the order of study enrollment. The allocation of dyads compared to non-dyads did not significantly differ across the two writing conditions, *F*(1, 37) = 2.24, *p* = .143, indicating that randomization was successful. Efforts to reduce experimenter bias included distributing participants’ writing instructions in a sealed folder so that the experimenter was unaware of the writing condition and using multiple research assistants who were unaware of participants’ study condition to administer baseline and follow up assessments.

### Procedure

The study intervention involved patient and caregiver completion of 3, 20-minute writing sessions at an interdisciplinary movement disorders clinic in a private room or at home for individuals who lived greater than 1 h away from the center. The writing instructions [37] given to the expressive and neutral conditions at each of the three writing sessions are below:

#### Instructions for expressive writing condition

*For the next 3 days, I would like you to write your very deepest thoughts and feelings about the most traumatic experience of your entire life or an extremely important emotional issue that has affected you and your life. In your writing, I’d like you to really let go and explore your deepest emotions and thoughts. You might tie your topic to your relationships with others, including parents, lovers, friends or relatives; to your past, your present or your future; or to who you have been, who you would like to be or who you are now. You may write about the same general issues or experiences on all days of writing or about different topics each day. All of your writing will be completely confidential. Don’t worry about spelling, grammar or sentence structure. The only rule is that once you begin writing, you continue until the time is up.*

#### Instructions for neutral writing condition

*What I would like you to write about over the next 3 days is how you use your time. In your writing, I want you to be as objective as possible. I am not interested in your emotions or opinions. Rather I want you to try to be completely objective. Feel free to be as detailed as possible. In today’s writing, I want you to describe what you did yesterday from the time you got up until the time you went to bed. For example, you might start when your alarm went off and you got out of bed. You could include the things you ate, where you went, which buildings or objects you passed by as you walked from place to place. The most important thing in your writing, however, is for you to describe your days as accurately and as objectively as possible. All of your writing will be completely confidential. Don’t worry about spelling, grammar or sentence structure. The only rule is that once you begin writing, you continue until the time is up.*

Accommodations were made to ensure that participants at all levels of motor functioning were able to participate. Participants (*N* = 6) who were unable to handwrite all selected to type into a secure word-processing document on a computer, and these individuals were equally divided across the two writing conditions.

### Outcome measures

Outcome measures consisted of self-reported emotional functioning, QOL, performance on a brief battery of cognitive tests, and stress hormone levels. These outcomes were assessed prior to the first writing session (baseline), shortly after the third writing session (immediate post, mean follow up = 5.00 days), and at four (4-month follow up, mean follow up = 123.86 days) and 10 month follow-up (10-month follow up, mean follow up = 308.61 days) appointments. Self-reported outcomes were collected, managed, and stored in a secure web-based database (Research Electronic Data Capture (REDCap) [21]. The stress hormone levels were self-collected at home and then hand delivered for freezer storage. Specific measures and details of the stress hormone collection are described in detail below.

#### Self-report measures

A compilation of validated self-report questionnaires were used to assess various aspects of emotional functioning among the patient and caregiver participants and established clinical guidelines were used to interpret all scores. Cronbach’s alpha was calculated for each measure within the full study sample at baseline to assess internal reliability of these measures in our sample, with the following rule of thumb used for interpretation of alpha values: α ≥ 0.9 Excellent, 0.9 > α ≥ 0.8 Good, 0.8 > α ≥ 0.7 Acceptable, 0.7 > α ≥ 0.6 Questionable, 0.6 > α ≥ 0.5 Poor, 0.5 < α Unacceptable [18]. All self-report measures were found to have at least acceptable internal consistency in our sample.

All participants completed the *Multidimensional Anxiety Questionnaire* (MAQ; [41], a 40-item self-report instrument that captures anxiety within four domains (Physiological-Panic, Social Phobia, Worry-Fears, and Negative Affectivity) as well as an overall Total Score. The MAQ was selected to allow for differentiation of physical, cognitive, and emotional anxiety symptoms, given potential for elevated physical symptoms associated with PD. Good reliability and validity have been demonstrated for the MAQ, and separate norms are available for community adults and college students [41]. Community adult norms were used within this study, and established clinical cutoffs were relied upon for interpretation of scores. Cronbach’s alpha for the MAQ was calculated in our sample with, α = .91, indicating excellent internal consistency.

The *Beck Depression Inventory-II* (BDI-II; [3], a 21-item self-report questionnaire was used to capture the severity of depression symptoms for patients and caregivers. Strong psychometric properties have been established for the BDI-II, and it has been demonstrated to be a reliable measure of depression in older adult samples [17]. Cronbach’s alpha for the BDI was calculated in our sample with, α = .83, indicating good internal consistency.

The *Apathy Scale* (AS; [49] is a 14-item scale that was developed and validated to screen for apathy symptoms in individuals with PD. For caregivers, a modified version of item three was administered, which directed caregivers to consider their current condition, including social, financial, familial, physical, or emotional aspects of their functioning. Cronbach’s alpha for the AS was calculated in our sample with, α = .74, indicating acceptable internal consistency.

All participants also completed the *Linear Analogue Self Assessment* (LASA; [32], which assesses dimensions of physical, emotional, spiritual, intellectual, and overall QOL using single items. A recent needs assessment survey used this instrument to capture overall QOL in a sample of high functioning PD patients [31]. Cronbach’s alpha was not calculated for this scale because interpretation is done at the single-item level.

Daily cognitive functioning was measured in both patients and caregivers with the *Everyday Cognition* scale (ECOG; [14]), which provides a total score as well as six subscales measuring memory, language, visuospatial abilities, and planning, organization, and divided attention subdomains of executive functioning. This scale was validated in a large sample of older adults whose cognitive function ranged from healthy to demented [14]. Cronbach’s alpha for the ECOG total was calculated in our sample with, α = .98, indicating excellent internal consistency.

Patient specific measures included the *Parkinson’s Disease Non-Motor Symptom Questionnaire* (PD-NMS; [7], a 30-item questionnaire developed to assess the frequency of common non-motor symptoms in individuals with PD, and the *Parkinson’s Disease Questionnaire-39* (PDQ-39; [27], a 39-item self-report questionnaire which evaluates the impact of PD on QOL across eight domains, including Bodily Discomfort, Mobility, Activities of Daily Living, Emotional Well Being, Communication, Cognitive Impairment, Stigma, and Social Support. Both the PD-NMS and the PDQ-39 have been well validated in PD samples to capture non-motor symptomatology and health-related QOL, respectively. Cronbach’s alpha for the PD-NMS was calculated in our sample with, α = .78, indicating acceptable internal consistency.

Finally, caregiver burden was assessed using the 12-item short form of the *Zarit Burden Inventory* (ZBI; [4], which assesses the impact of caregiving on the caregiver’s emotional and physical health, as well as their ability to engage in social activities. Adequate psychometric properties of the short-form ZBI were demonstrated in a large sample of caregivers of cognitively impaired older adults and are likely to generalize to other caregivers of community dwelling older adults. Cronbach’s alpha for the ZBI was calculated in our sample with, α = .76, indicating acceptable internal consistency.

#### Cognitive performance measures

Patient and caregiver participants completed a brief battery of neurocognitive measures including tests assessing processing speed and mental flexibility (Trailmaking Tests A & B [23, 40], auditory attention and working memory (Digit Span (DS) and Letter Number Sequencing (LNS) [11, 53]; Auditory Consonant Trigrams (ACT) [6, 38] and verbal learning and memory (California Verbal Learning Test – II (CVLT) [11]).

#### Writing theme code book

Writing samples from the expressive writing condition were reviewed, coded, and tested for inter-rater reliability in order to be able to optimally categorize the writing topics. It was initially hypothesized that a majority of the participants in the expressive condition would select PD diagnosis as their writing topic, given salience of PD as a major medical illness in their lives. However, when the writing samples were reviewed, PD diagnosis was selected as a writing topic by only one patient out of 11 and none of the caregivers. Because the number of participants that selected PD diagnosis as their writing topic was not sufficient, a wider range of writing topics was taken into consideration. A codebook of categorical writing topics was established by the research team. Two researchers (TVC and SKL) independently rated the writing topics in order to establish inter-rater reliability of the codebook categories, which was measured by Cohen’s kappa coefficient. Cohen’s kappa coefficient (κ = 0.93) indicated strong inter-rater reliability for the codebook categories.

#### Stress hormone measurement

The CAR has been shown to be a valuable measure for assessing hyper- and hypocortisolemic traits in patients with depression and chronic fatigue [24]. Specifically, overall waking cortisol level, known as AUCt, can provide reliable trait-level information about the activity level of the HPA axis [24]. In line with established reliability criteria [24], AUCt levels were assessed using saliva samples collected on 2 weekday mornings immediately after waking, as well as 30, 45 and 60 min after the waking time at each of the four timepoints (Baseline, Immediate Post, 4-Month, 10-Month). A total of 32 samples over the course of the study (four samples on eight different mornings) were self-collected by participants at home using a salivette device. Participants were asked to record sample collection times in a provided log as well as on the sample tubes to ensure that data was collected at the correct times. Subjects were asked to store the salivettes in the freezer and to deliver them to our clinic immediately after collecting the last consecutive sample. In the center-associated laboratory, the saliva samples were centrifuged and stored at −20°C until analyzed using salivary cortisol analysis performed by the Center for Clinical & Translation Research’s Clinical Research Services Unit Laboratory utilizing a sandwich immunoassay methodology supplied by Salimetrics, LLC. To our knowledge, the CAR has not been evaluated previously among individuals with PD or their caregivers. In one prior study [39], the CAR was captured in a sample of healthy older adults, and levels peaked at 30 min post awakening, with an average of .91 μg/dL at that time point. Assessment of the CAR among Alzheimer’s patients and their caregivers revealed elevations at 30 min post awakening, with average levels of 1.20 μg/dL for caregivers and 1.30 μg/dL for patients [52]. For analyses in this study, salivary cortisol levels at waking, and 30, 45, and 60 min post waking across the two collection days were averaged at each study time point, and these values are presented in Table 1, with higher values indicating greater cortisol reactivity.

**Table 1:** Non-motor, cognitive, and physiological outcomes means, standard errors, and effects by writing condition and timepoint

	Expressive writing				Neutral writing							
Outcome mean (SE)	Pre	Post	4 M	10 M	Pre	Post	4 M	10 M	Effects	*F* value	*p* value	*η* ^*2*^
Self-reported non-motor functioning												
MAQ	48.16 (2.35)	46.86 (2.03)	45.48 (2.14)	46.51 (1.99)	48.11 (2.48)	45.90 (2.15)	43.94 (2.26)	44.18 (2.10)	Time	3.65	.016*	.13
									Time X Condition	.40	.75	.02
BDI-II	6.70 (1.46)	4.53 (.98)	5.99 (1.27)	6.03 (1.10)	6.20 (1.54)	4.66 (1.04)	4.65 (1.34)	5.03 (1.16)	Time	2.53	.064**	.09
									Time X Condition	.45	.720	.02
AS	9.66 (1.12)	10.35 (1.25)	10.76 (1.27)	11.06 (1.37)	8.98 (1.18)	6.94 (1.32)	8.10 (1.34)	8.60 (1.44)	Time	.76	.521	.03
									Time X Condition	1.03	.383	.04
LASA	8.19 (.40)	8.63 (.36)	8.53 (.53)	7.89 (.52)	7.95 (.42)	8.31 (.38)	8.41 (.55)	7.68 (.55)	Time	2.01	.120	.07
									Time X Condition	.17	.916	.01
ECOG	66.03 (5.75)	57.97 (2.95)	61.05 (4.19)	57.88 (4.31)	51.63 (6.82)	48.81 (3.50)	48.94 (4.97)	51.31 (5.12)	Time	1.94	.132	.08
									Time X Condition	1.02	.387	.04
PD-NMS (*N* = 19)	6.73 (1.34)	7.46 (1.04)	6.55 (1.35)	8.27 (1.35)	9.88 (1.57)	8.00 (1.22)	8.75 (1.59)	10.38 (1.58)	Time	2.02	.122	.11
									Time X Condition	.98	.408	.06
PDQ-39 (*N* = 19)	18.73 (5.16)	20.46 (5.47)	20.64 (4.70)	23.91 (5.40)	23.75 (6.06)	20.63 (4.42)	23.00 (5.51)	22.63 (6.33)	Time	.55	.652	.03
									Time X Condition	.77	.516	.04
ZBI (*N* = 10)	7.20 (2.00)	5.20 (1.45)	7.40 (2.03)	7.00 (2.02)	7.40 (2.00)	4.40 (1.45)	4.20 (2.03)	4.80 (2.02)	Time	1.88	.160	.19
									Time X Condition	1.00	.409	.11
Cognitive performance												
Trails A	47.06 (2.88)	50.85 (2.91)	52.71 (2.49)	52.33 (3.23)	50.60 (2.82)	51.09 (2.84)	50.58 (2.43)	52.19 (3.16)	Time	1.52	.218	.06
									Time X Condition	.94	.426	.04
Trails B	51.64 (2.57)	54.25 (3.16)	54.74 (3.58)	52.03 (3.74)	51.14 (2.51)	51.85 (3.09)	54.95 (3.49)	57.13 (3.65)	Time	2.14	.102	.08
									Time X Condition	2.14	.102	.08
ACT	46.42 (1.59)	47.91 (1.69)	48.23 (1.72)	48.63 (1.95)	44.75 (1.56)	47.76 (1.65)	46.58 (1.68)	48.20 (1.91)	Time	2.58	.060**	.10
									Time X Condition	.56	.642	.02
DS	11.49 (.68)	10.69 (.69)	11.75 (.65)	11.99 (.63)	12.00 (.66)	12.36 (.68)	11.50 (.63)	11.78 (.62)	Time	.28	.838	.01
									Time X Condition	2.43	.072**	.09
LNS (*N* = 18)	11.70 (.72)	12.27 (1.01)	11.88 (.73)	11.73 (.84)	8.70 (.95)	10.75 (1.35)	9.80 (.97)	9.35 (1.12)	Time	1.56	.214	.10
									Time X Condition	.48	.700	.03
CVLT-II												
Trials 1–5 T	55.92 (3.84)	58.32 (3.33)	57.46 (3.62)	60.15 (3.38)	53.53 (3.75)	57.24 (3.25)	58.93 (3.54)	58.70 (3.30)	Time	2.86	.043*	.11
									Time X Condition	.49	.691	.02
CVLT-II Short Delay Free Recall	.17 (.36)	.44 (.35)	.48 (.45)	.31 (.35)	-.21 (.35)	.54 (.34)	.38 (.44)	.86 (.34)	Time	3.54	.019*	.13
									Time X Condition	1.91	.135	.07
CVLT-II Long Delay Free Recall	-.14 (.35)	.40 (.30)	.28 (.41)	.39 (.34)	.10 (.34)	.69 (.29)	.51 (.40)	.89 (.33)	Time	5.76	.001*	.19
									Time X Condition	.28	.841	.01
CVLT-II Recognition	-.11 (.36)	.22 (.33)	.38 (.37)	.44 (.35)	.23 (.35)	.09 (.32)	.54 (.36)	.50 (.35)	Time	2.58	.060**	.10
									Time X Condition	.56	.642	.02
Salivary cortisol (μg/dL, values represent mean levels across 60 min post waking)									Type III Tests of Fixed Effects	*F* value	*p*	
Full sample (*N* = 25)	.33 (.11)	.29 (.11)	.35 (.11)	.40 (.11)	.36 (.11)	.54 (.12)	.64 (.12)	.65 (.13)	Time	1.08	.366	
									Time X Condition	.88	.456	
									Time X Condition X Status	2.78	.049*	
Patients (*N* = 16)	.28 (.12)	.23 (.12)	.32 (.13)	.29 (.12)	.37 (.13)	.73 (.13)	.43 (.14)	.63 (.15)	Time	1.31	.288	
									Time X Condition	2.62	.067**	
Caregivers, (*N* = 9)	.38 (.20)	.34 (.20)	.37 (.20)	.50 (.20)	.34 (.20)	.35 (.20)	.84 (.22)	.66 (.22)	Time	1.34	.287	
									Time X Condition	1.24	.319	

*N* = 29 for all analyses unless otherwise specified

Alpha level is designated as *p <.05, and **p <.10 designates a marginally significant trend

*η* ^*2*^ = proportion of variance accounted for by each effect (e.g., .31 = 31 %)

Interpretation of *η* ^*2*^ effect sizes should be as follows: ≥ .02 = small effect, ≥ .13 = medium effect, ≥ .26 = large effect

Outcome abbreviations are as follows: *MAQ* multidimensional anxiety questionnaire, *BDI-II* Beck Depression Inventory-II, *AS* apathy scale, *LASA* linear analogue self assessment, *ECOG* everyday cognition scale, *PD-NMS* Parkinson’s disease non-motor symptom questionnaire, *PDQ-39* Parkinson’s disease quality of life-39, *ZBI* Zarit Burden inventory, *ACT* auditory consonant trigrams, *DS* digit span, *LNS* letter number sequencing, *CVLT-II* California verbal learning test-II, *CAR* cortisol awakening response

## Results

### Feasibility

From an initial sample of eligible individuals with PD (*N* = 27) and caregivers (*N* = 14) who consented to participate in the study, 15 patients and eight caregivers were randomly assigned to the expressive writing condition and 12 patients and six caregivers were assigned to the neutral writing condition. Eight patients (29.62 %; four randomized to expressive writing and four randomized to neutral writing) and four caregivers (28.57 %; two randomized to expressive writing and two randomized to neutral writing) chose to discontinue before beginning the intervention or were lost to follow up prior to completing the intervention or the first follow up visit. There were no significant differences in attrition between the two conditions, and participants cited not wanting to disclose personal information, travel distance to the clinic for multiple follow up appointments, and new medical symptoms unrelated to the study intervention as reasons for discontinuing. The final sample included in the analyses were those who completed all sessions of neutral or expressive writing and completed at least baseline and immediate post follow-up visits (*N* = 19 patients (11 = expressive writing, 8 = neutral writing), ten caregivers (6 = expressive writing, 4 = neutral writing). Participants were permitted to participate with or without providing cortisol samples; therefore the sample size is reduced for the cortisol analyses (*N* = 16 patients, nine caregivers), with no significant differences in willingness to provide cortisol samples observed between the two writing conditions.

### Writing themes

A descriptive analysis was performed to determine the percentage of patients that chose each writing topic. Qualitative review of the expressive writing condition essays using established guidelines for content analysis of expressive writing transcripts [15] resulted in a codebook with eight topic categories, including family relationships, life-threatening event, death, PD diagnosis, illness of a family member, life review, self-health problems/illness, and reflection on the purpose of the expressive writing exercise. Qualitative review of the neutral writing condition essays was also performed as a manipulation check to ensure that participants assigned to this condition did not write about emotional topics despite instructions to write descriptively. All neutral writing condition essays were judged by two reviewers and were determined to conform to study instructions; therefore, no further analyses were performed on the neutral essays. Frequency analyses were performed to determine which codebook categories were used most frequently by patients and caregivers. The largest percentage of the participants focused on “Family Relationships” (36.4 %) as their writing topic, followed by “Death” (25.5 %), “Life-threatening Event” (12.7 %), and “Illness of a Family Member” (9.1 %). Only 1.8 % of patients and caregivers in the expressive writing condition wrote about the patient’s PD diagnosis as their most stressful life experience.

### Self-reported non-motor functioning

Overall, individuals with PD endorsed an average level of eight non-motor symptoms (e.g., mood/anxiety symptoms, cognitive impairments, gastrointestinal problems, sleep disturbances) consistent with prior research [7], non-clinically significant levels of anxiety, depression, and apathy, and positive QOL. Caregivers also reported non-clinical levels of mental health symptoms, positive QOL, and low caregiver burden. See Table 1 for means and standard errors at baseline and across the four study timepoints. Repeated measures analyses of variance (ANOVA) using a 2 Writing Condition (traumatic, neutral) x 2 Participant Status (patient, caregiver) x 4 Timepoint (Baseline, Immediate Post, 4-Month, 10-Month) design were performed for each of the self-reported outcomes to determine effects on non-motor symptoms, overall and health-related QOL, anxiety, depression, and caregiver burden. Given the assumption that the unit of analysis for repeated measures ANOVA is the subject and not the group, analyses were initially performed with dyad status (coded as dyad or non-dyad) as an additional between subjects variable to determine whether participation in pairs affected the results. Dyad status did not have a significant effect on any self-report outcomes (results not included in this report); therefore, we proceeded with analyses on the mixed sample of dyadic and non-dyadic caregivers and patients. Patients and caregivers did not significantly differ in their self-reported anxiety, apathy, overall QOL, or everyday cognitive functioning at baseline; however, significant differences were detected in depression, such that patients reported significantly higher depression symptoms on the BDI-II at baseline than caregivers, *F*(1,39) = 6.44, *p* = .015. Therefore, the total sample of patients and caregivers was analyzed together for optimal statistical power, but the effect of participant status was included in all models to account for any differences between the two samples. No baseline differences between the expressive and neutral writing conditions were found. No significant differences were detected between participants who handwrote compared to those who wrote on a computer; therefore, analyses were performed on the combined sample.

See Table 1 for means and standard errors separated by writing condition across the four study time points. Appropriate *F* values, significance levels, and effect sizes for the main effect of time and interaction of time and writing condition are also included in Table 1. Significant and marginally significant effects are highlighted below. The effect size value partial eta squared (*η*^2^) captures the proportion of variance accounted for by each effect (e.g., .31 = 31 %). Interpretation of *η*^2^ effect sizes should be as follows: ≥ .02 = small effect, ≥ .13 = medium effect, ≥ .26 = large effect.

Anxiety symptoms (MAQ) significantly declined following the intervention, *F*(3, 75) = 3.65, *p* = .016, *η*^*2*^ = .13, with no significant differences between the two writing groups. Depression symptoms (BDI-II) showed a marginally significant trend for decreasing over time in both writing conditions, *F*(3, 75) = 2.53, *p* = .064, *η*^*2*^ = .09, with a significant reduction from baseline to immediate post-test, *F*(1,25) = 5.57, *p* = .026, *η*^*2*^ = .18. The overall effects of Timepoint and the interaction of Timepoint X Condition were not significant for self-reported everyday cognitive functioning (ECOG); however, a significant Timepoint X Condition interaction was observed from the 4-Month to the 10-Month follow up, with improvement in cognitive functioning for expressive writers and the opposite pattern for neutral writers, *F*(1, 24) = 7.51, *p* = .011, *η*^*2*^ = .24. Within the caregiver sample, caregiver burden (ZBI) did not show the anticipated main effect of time or interaction of time and writing condition, but there was a marginally significant quadratic trend, *F*(1,8) = 4.49, *p* = .067, *η*^*2*^ = .36, with a significant reduction in burden from baseline to immediate post, *F*(1, 8) = 5.95, *p* = .041, *η*^*2*^ = .43. No significant or marginally significant effects of Writing Condition, Participant Status, or Timepoint were observed on other self-report outcome measures, which included PD-related QOL (PDQ-39), non-motor symptoms (PD-NMS), apathy symptoms (Apathy Scale), and overall QOL (LASA), all *p*s >.10.

### Cognitive functioning

Baseline cognitive functioning did not differ significantly between caregivers and patients on main cognitive outcome measures; therefore repeated measures ANOVAs were performed on the combined sample to assess the effects of Participant Status, study Timepoint, and Writing Condition on cognitive outcomes with optimal statistical power. No baseline differences between the expressive and neutral writing conditions were found. As noted above, dyad status was evaluated as a potential factor that could have affected the repeated measures ANOVA results, but no significant effects of dyad status were found on cognitive outcomes (results not included in this report).

Performance on tasks of verbal learning (CVLT Trials 1–5), *F*(3,72) = 2.86, *p* = .043, *η*^*2*^ = .11 and memory (CVLT Short Delay Free Recall), *F*(3, 72) = 3.54, *p* = .019, *η*^*2*^ = .13; (CVLT Long Delay Free Recall), *F*(3, 72) = 5.76, *p* = .001, *η*^*2*^ = .19 significantly improved over time, but no differences were found between the two writing conditions. No significant changes were found in participants’ performance on tasks measuring processing speed (Trails A), processing speed and mental flexibility (Auditory Consonant Trigrams and Trails B), and auditory attention and working memory (Digit Span). A marginally significant effect of Timepoint was found on a mental flexibility task (ACT) such that performance improved over time in both writing groups, *F*(3,72) = 2.58, *p* = .060, *η*^*2*^ = .10. Finally, a marginally significant interaction of Timepoint and Writing Condition was seen on a test of auditory attention and working memory (Digit Span), with slight overall improvement seen for those in the expressive writing condition compared to the neutral writing condition, *F*(3,72) = 2.43, *p* = .072, *η*^*2*^ = .09.

### Cortisol awakening response

Sample collection time logs revealed participants were compliant with salivary collection procedures. Multilevel modeling was used to analyze the fixed effects of Writing Condition and Participant Status and random effects of study Timepoint on the CAR. No baseline differences between the expressive and neutral writing conditions or between patients and caregivers were found. Dyad status was initially included in the model as an additional fixed effect to determine whether those participating in pairs differed in their cortisol response compared to those participating alone. No significant effect of dyad status was found (results not included in this report).

A significant three-way interaction of Participant Status x Condition X Timepoint, *F*(3, 52.94) = 2.78, *p =* .049, was observed*.* When evaluated separately for patients and caregivers, the interaction of Condition X Timepoint trended towards significance for patients, *F*(3, 33.58) = 2.62, *p* = .067, but not for caregivers, *p* = .319. CAR was lower for individuals with PD in the expressive writing condition at immediate-post and 10-month follow-up compared to those in the neutral writing condition.

## Discussion

This study is the first to examine the feasibility and potential benefits of an expressive writing intervention for individuals with PD and their caregivers. Attrition rates, a variety of self-report outcomes, performance on tests of neurocognitive functioning, and an objective stress hormone response measure were assessed to determine the feasibility and effect size of an expressive writing intervention in PD patient and caregiver samples.

Although some positive findings were observed, the majority of outcome variables were non-significant. In a pilot study, small sample size can often limit conclusions that can be made. While the positive results are encouraging, experience of significant study recruitment and retention issues were challenging. Specifically, nearly one third of consented participants failed to complete the study protocol. The majority of these individuals were lost to follow up and failed to return multiple contact attempts by study personnel. Of those who provided a reason for discontinuing, travel distance, hesitation to engage in personal disclosure, and the emergence of new medical symptoms unrelated to study participation were reported. Most participants agreed to provide salivary cortisol samples, but a small number chose not to do so. Attrition rates and willingness to provide salivary cortisol samples did not differ systematically between expressive and neutral writing conditions.

Overall, these results indicate that a brief expressive writing intervention and collection of salivary samples was tolerated by most participants and that study condition did not influence willingness to participate in the study. These findings suggest that a larger scale trial would likely be successful in recruiting and retaining a sufficient sample, but that additional efforts may be needed to reduce attrition to recruit necessary numbers of participants in a reasonable timeframe. The length of follow up and at home collection of cortisol samples in this study may have been burdensome to participants. Future studies might include fewer follow up visits, particularly given that most effects of the intervention were detected at immediately post intervention. Collection of cortisol samples immediately before and after writing to detect state-level shifts in cortisol reactivity might reduce participant burden and provide additional insight into the physiological effects of expressive writing.

Another unanticipated finding of this pilot study included that most of the participants, including both patients and caregivers, did not choose a PD-related topic for their expressive writing. The most frequently chosen topics involved themes such as family relationships, death, and life-threatening events. This finding suggests that in this sample the most stressful factors in the patients’ and caregivers’ lives were not PD-related and tend to be common life events that are not particularly focused on PD. Awareness that general life stressors and their impact on individuals with PD and their caregivers may be of more concern than PD-specific issues is critical for health care providers as well as the general public to recognize when helping support and provide care for individuals with PD and their caregivers. It remains unclear if common life stressors have additional impacts on individuals’ motor and non-motor symptoms, but this certainly seems plausible. Consideration of potential impact of common life stressors on emotional and physical health is recommended to provide individuals with PD and their caregivers more effective, efficient and comprehensive treatment plans.

Importantly, in the evaluation of self-reported outcomes, results showed positive changes over time on measures of anxiety, depression, and caregiver burden in both the expressive and neutral writing conditions. Improvement of emotional functioning in a neurodegenerative disease with known impact on dopamine levels demonstrates potential promise of biopsychosocial interventions in this population. Superior improvement seen within the expressive writing condition on a measure of self-reported everyday cognitive functioning is also encouraging but should be replicated, given that improvements were only observed during the follow up period. No reliable changes were observed on measures of apathy, PD-related or overall QOL, or non-motor symptoms. One explanation for the lack of change on these measures could be the low levels of apathy and non-motor symptoms and high QOL reported within this sample.

Indeed, prior research confirms that individuals with mild to moderate distress at baseline are most likely to benefit from expressive writing [34]. Less robust benefit from the intervention observed in our sample may have been due to the non-clinical levels of emotional symptoms reported by participants. In addition, improvements took place in both writing conditions, suggesting that involvement in a research study and perhaps especially engagement in writing about both emotional and neutral topics may serve as a buffer against the progression of emotional symptoms in individuals with PD and their caregivers. Several previous studies have shown a lack of difference in outcomes across writing conditions in caregivers [33, 43] and patient [42] samples, particularly on psychological health outcomes. While the reasons for these discrepant findings remain unclear, our results suggest that a brief, psychosocial intervention has benefits on select emotional outcomes for individuals with PD and their caregivers.

With regard to cognitive functioning, verbal learning and memory improvements were observed independent of writing condition. This finding is not likely due to practice effects given use of alternate forms and largest impact of repeated exposure to cognitive tasks between the first and second exposure [26]. Learning and memory retrieval problems are commonly observed in individuals with PD and persons experiencing significant life stressors [51]. Experience of these symptoms can have negative impact on work functioning and independent living. Cognitive rehabilitation techniques have increasingly been studied in individuals suffering from Mild Cognitive Impairment (MCI), with mixed but some promising results [20, 25], but have not yet been explored in individuals with PD. We are currently completing two trials examining in-person and computerized cognitive rehabilitation strategies in individuals with PD-MCI and initial results show some positive impact [29, 30]. Further study of behavioral treatments for memory issues is relevant for both medical and healthy populations given the aging of our population. These findings highlight the possibility that emotional and cognitive functioning may interact in both individuals with PD and their caregivers.

Results examining the effects of the writing conditions and participant status over time on the CAR were also encouraging. The hypothesis that expressive writing would lead to lower CAR over the course of the study was partially supported among individuals with PD. However, caregiver participants did not show the expected decline in CAR. In patients, the pattern of change in CAR over time was indicative of increased CAR for those in the neutral writing condition, with a more stable trajectory for those in the expressive writing condition. This pattern suggests a possible buffer effect of expressive writing compared to neutral writing on rising cortisol levels in individuals with PD. It is also important to note that, although there was a significant interaction of participant status, writing condition, and time, the effect was trending towards significance but did not reach significance when examined within the patient sample.

### Limitations

These findings should be interpreted in the context of several study limitations. The sample size was somewhat low, although not substantially lower than other pilot studies of expressive writing in other chronically ill populations [9, 42]. Low sample size limits statistical power and the ability to detect statistically significant effects. However, the fact that we were able to detect several significant effects, even in our limited sample size, speaks to the potential efficacy of this intervention. A second limitation is that several of our measures have not been previously used in individuals with PD or among caregivers. As such, interpretation of the MAQ and CAR results were guided by literature on related samples and should be replicated in future research. In addition, we did not control for medication usage, including dosage amounts of levodopa medication commonly used to treat PD and steroid medications, which may have influenced cortisol findings. Finally, our sample was fairly homogenous in terms of racial/ethnic background, educational attainment, and functional status, and results may not generalize to more diverse or more impaired groups of PD patients and caregivers. Inclusion of a wider variety of individuals should be prioritized in future studies to determine whether writing interventions are useful to PD patients and caregivers from different backgrounds and with more significant functional impairments.

## Conclusions

This pilot study provides initial findings regarding the feasibility and effectiveness of a brief, cost-effective psychosocial intervention among individuals with PD and their caregivers. The study demonstrated moderate feasibility but questionable effectiveness for expressive writing in a small PD patient and caregiver sample. No significant differences between the expressive and neutral writing conditions were found on emotional and cognitive outcome variables. However, positive changes over time on measures of anxiety, depression, and caregiver burden were observed in both the expressive and neutral writing conditions, verbal learning and memory improvements were observed independent of writing condition, and the hypothesis that expressive writing would lead to lower CAR over the course of the study was supported, but only among individuals with PD.

Low recruitment rates (40.9 % of eligible participants chose to participate) and fairly high attrition rates (29.3 % of all participants dropped out before study completion) were challenges encountered in this study. Individuals who declined participation cited travel distance or lack of interest as primary reasons for not completing the study, suggesting that telehealth or internet-based protocols for expressive writing may increase recruitment rates. Attrition rates were largely explained by changes in participants’ life circumstances, whether related to the emergence of new medical symptoms unrelated to the study or other life obligations that prevented travel to the clinic to complete the study. Given the older age of our targeted sample, it is not surprising that new medical problems were a barrier to completing the study. As with recruitment challenges, one solution to the barrier of traveling to our clinic is to offer telehealth or internet-based protocols for future interventions. A final consideration is that, as acknowledged in our informed consent process with participants, writing about an emotionally stressful life experience can result in a temporary increase in negative emotions and physiological arousal (typically followed by a larger decrease in these symptoms). As such, some participants or potential participants may have declined to participate or dropped out of the study to avoid this temporary discomfort, leading to reduced recruitment rates and/or increased attrition.

Changes that occurred in our sample may be attributable to the passage of time and/or the process of writing, whether about a neutral or an emotionally salient topic, which is consistent with some prior expressive writing studies, which also did not detect differences between the writing conditions [36]. Expressive writing was compared to neutral writing in order to be consistent with prior research, based on the assumption that writing about an emotional topic promotes therapeutic change [45], while writing about a neutral topic has not been theorized to promote clinically meaningful changes. However, a no-writing control group may be a useful addition in future studies in order to better control for potential confounds (e.g., maturation) as well as potential benefits associated with the writing process itself.

One exception to the pattern of null results in our study was the three-way interaction of participant status, writing condition, and time on the CAR, with lower cortisol reactivity seen for PD patients across time in the expressive writing condition. However, these results are tentative given the small sample and the lack of significant effects within the PD sample. It may be that three sessions of emotional writing were not sufficient to produce statistically reliable and clinically significant change in cortisol reactivity and psychological distress for individuals with PD and their caregivers. It is possible that additional writing sessions would be necessary to produce more marked changes in this population, given the complexity of motor and non-motor symptoms in PD.

Future work in this area should explore the specific conditions of writing that can enhance its benefits, especially among individuals with PD and other movement disorders and their caregivers, for whom very few empirically evaluated alternative and complementary interventions are currently available. Further qualitative examination of patients’ and caregivers’ written narratives is planned and will facilitate a test of theories of expressive writing as well as generate new explanations for its mechanisms of action within a novel population. In sum, this study is an important first step towards empirical evaluation of a well-established psychosocial intervention within an underserved population, and ongoing efforts of this kind are likely to substantially contribute to the physical and emotional well-being of individuals with PD and their caregivers.

## Additional file

Additional file 1:
**Figure S1.** CONSORT flow diagram for expressive writing pilot in individuals with PD and their caregivers I have added the citation in the main body of the text referencing the figure. (DOCX 73 kb)

## Abbreviations

ACT: Auditory consonant trigrams
AS: Apathy scale
BDI-II: Beck Depression Inventory-II
CAR: Cortisol awakening response
CBT: Cognitive-behavioral therapy
CVLT: California verbal learning test-II
DS: Digit span
ECOG: Everyday cognition scale
LASA: Linear analogue self assessment
LNS: Letter number sequencing
MAQ: Multidimensional anxiety questionnaire
PD: Parkinson’s disease
PD-NMS: Parkinson’s disease non-motor symptom questionnaire
PDQ-39: Parkinson’s disease questionnaire-39
QOL: Quality of life
REDCap: Research electronic data capture
ZBI: Zarit Burden inventory
