# Supplementary material for: Randomized controlled expressive writing pilot in individuals with Parkinson’s disease and their caregivers
Source: BMC Psychol. 2015 Nov 30;3:44. doi: 10.1186/s40359-015-0101-4 (PMC4666161; doi:10.1186/s40359-015-0101-4)
Supplement: Additional file 1: — Figure S1. CONSORT flow diagram for expressive writing pilot in individuals with PD and their caregivers I have added the citation in the main body of the text referencing the figure. (DOCX 73 kb) [file 40359_2015_101_MOESM1_ESM.docx]

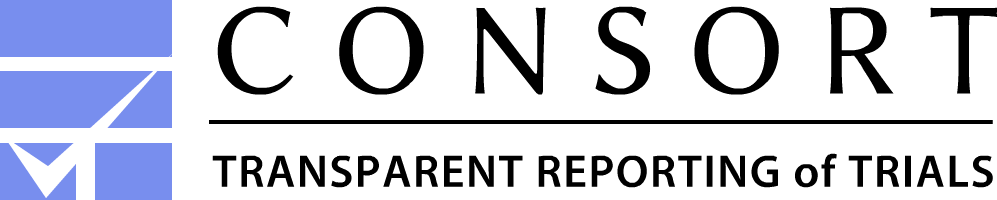


**CONSORT 2010 Flow Diagram**

## Allocation

Randomized (n= 27 patients, 14 caregivers)

Assessed for eligibility (n=66 patients)

Allocated to neutral writing

(n=12 patients, 6 caregivers)

♦ Received allocated intervention

(n=8 patients, 4 caregivers)

Provided salivary samples

(n=7 patients, 4 caregivers)

♦ Did not receive allocated intervention (give reasons) (n= )

## Follow-Up

Allocated to expressive writing

(n=15 patients, 8 caregivers)

♦ Received allocated intervention

(n=11 patients, 6 caregivers)

Provided salivary samples

(n=9 patients, 5 caregivers)

♦ Did not receive allocated intervention (give reasons) (n= )

Excluded (n= 39 patients)

♦  Not meeting inclusion criteria (n=0)

♦  Declined to participate (n=39)

♦  Other reasons (n=0)

## Enrollment

Analysed (n=11 patients, 6 caregivers for emotional outcomes; n=9 patients, 5 caregivers for cortisol outcomes)
♦ No participants were excluded from analyses

Analysed (n=8 patients, 4 caregivers for emotional outcomes; n=7 patients, 4 caregivers for cortisol outcomes)
♦ No participants were excluded from analyses

## Analysis

Lost to follow-up (did not return 3 or more attempts to contact) (n=3 patients, 2 caregivers)

Discontinued intervention (not comfortable writing about personal topics) (n=1 patient)

Lost to follow-up (did not return 3 or more attempts to contact) (n=3 patients)

Discontinued intervention (increased symptoms, travel distance) (n=1 patient, 2 caregivers)
